# Supplementary material for: Distinct EH domains of the endocytic TPLATE complex confer lipid and protein binding
Source: Nat Commun. 2021 May 24;12:3050. doi: 10.1038/s41467-021-23314-6 (PMC8144573; doi:10.1038/s41467-021-23314-6)
Supplement: Supplementary file 1 — Supplementary Information [file 41467_2021_23314_MOESM1_ESM.pdf]

Supplementary Information file

Distinct EH domains of the endocytic TPLATE complex confer lipid and protein binding.

Klaas Yperman[1,2], Anna C. Papageorgiou[3,*], Romain Merceron[4,5,*], Steven De Munck[4,5], Yehudi Bloch[4,5], Dominique Eeckhout[1,2], Qihang Jiang[1,2], Pieter Tack[6], Rosa Grigoryan[7], Thomas Evangelidis[3], Jelle Van Leene[1,2], Laszlo Vincze[6], Peter Vandenabeele[6,8], Frank Vanhaecke[7], Martin Potocký[9], Geert De Jaeger[1,2], Savvas N. Savvides[4,5,#], Konstantinos Tripsianes[3,#], Roman Pleskot[1,2,9,#] & Daniel Van Damme[1,2,#]

* Equal contribution.

# Corresponding authors. savvas.savvides@irc.vib-ugent.be (S.N.S), kostas.tripsianes@ceitec.muni.cz (K.T.), pleskot@ueb.cas.cz (R.P.), daniel.vandamme@psb.vib-ugent.be (D.V.D.).

[1]Ghent University, Department of Plant Biotechnology and Bioinformatics, Technologiepark 71, 9052 Ghent, Belgium.

[2]VIB Center for Plant Systems Biology, Technologiepark 71, 9052 Ghent, Belgium.

[3]CEITEC-Central European Institute of Technology, Masaryk University, Kamenice 5, 62500, Brno, Czech Republic.

[4]Department of Biochemistry and Microbiology, Ghent University, 9052 Ghent, Belgium.

[5]VIB Center for Inflammation Research, 9052 Ghent, Belgium.

[6]Department of Chemistry, X-ray Microspectroscopy and Imaging – XMI research unit, Ghent University, 9000 Ghent, Belgium

[7]Department of Chemistry, Atomic & Mass Spectrometry – A&MS research unit, Ghent University, Campus Sterre, Krijgslaan 281-S12, 9000 Ghent, Belgium.

[8]Archaeometry Research Group, Department of Archaeology, Ghent University, Sint-Pietersnieuwstraat 35, B-9000 Ghent, Belgium

[9]Institute of Experimental Botany, Academy of Sciences of the Czech Republic, Rozvojová 263, 16502 Prague 6, Czech Republic

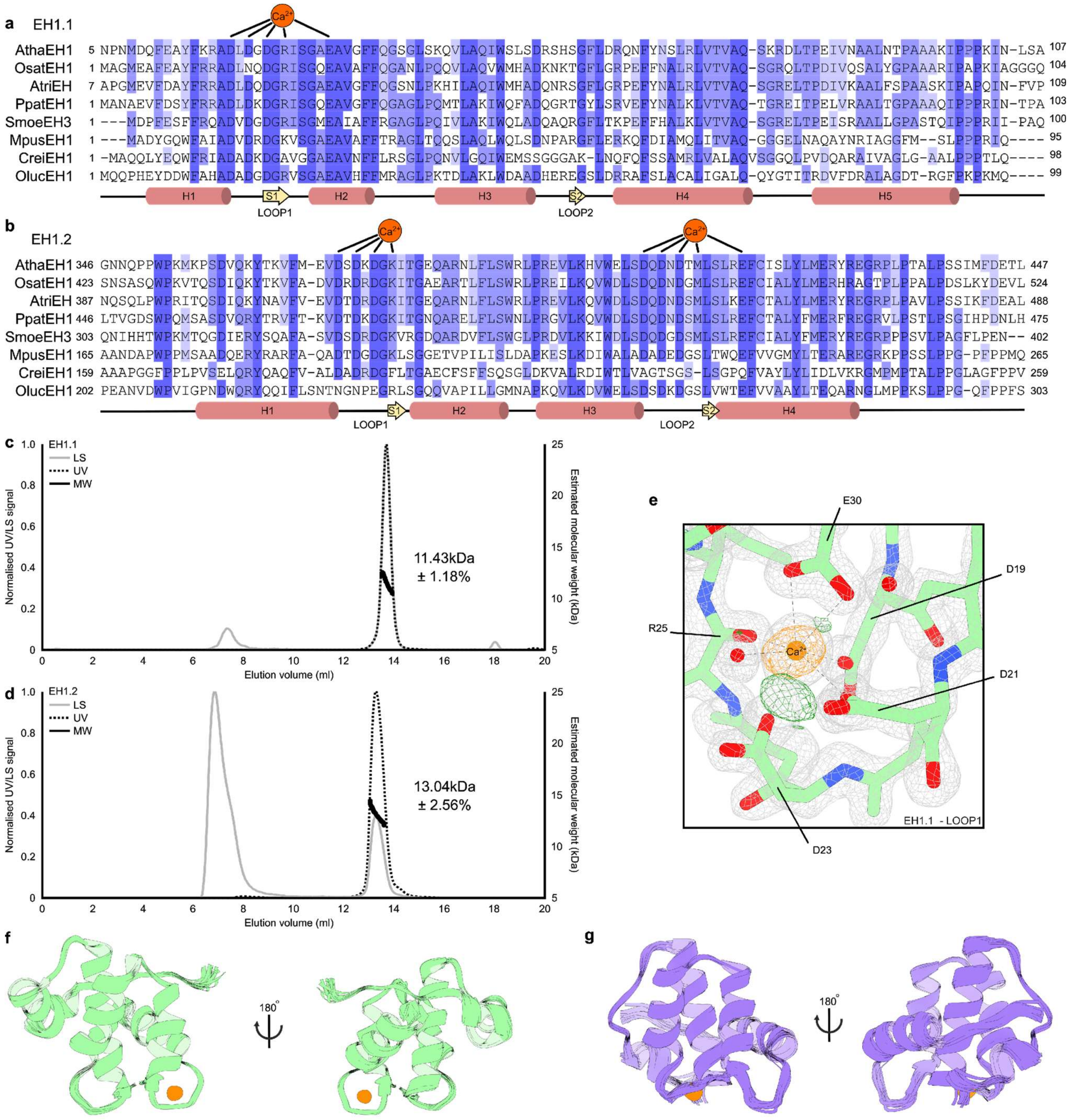

Supplementary Figure 1: Both EH domains of AtEH1/Pan1 show Ca^{2+} dependent reversible folding. a-b, Detail of the MSA of the first and second EH domain of AtEH1/Pan1 (EH1.1 and EH1.2) of a selected number of species. Ca^{2+} coordination is shown above and the secondary structural elements are indicated below each MSA. Atha - *Arabidopsis thaliana*, Osat - *Oryza sativa*, Atri - *Amborella trichocarpa*, Ppat - *Physcomitrella patens*, Smoe - *Selaginella moellendorffii*, Mpus - *Micromonas pusilla*, Crei - *Chlamydomonas reinhardtii*, Oluc - O*streococcus lucimarinus*. **c-d**, Size exclusion chromatography multi-angle laser light scattering elution profiles (Superdex 75 10/300), showing UV and light scattering signal (LS) of both EH domains of AtEH1/Pan1. The molecular weight distribution (MW) over the main peak is shown as black dots. **e**, Detail of the X-ray structure of the first EF-hand loop of EH1.1. The Fourier electron density map with coefficients 2mFo-DFc is shown as a grey mesh (contour level 2σ). Residual positive and negative difference electron density maps with coefficients mFo-DFc (contour level ±3σ)

are shown in green and red (none present), respectively. Anomalous difference Fourier electron density for Ca^{2+} is shown as an orange mesh (contour level 5σ). Side chains involved in Ca^{2+} (orange sphere) coordination are shown as sticks. **f-g**, The ensemble of the 20 lowest energy NMR structures of EH1.1 (green, 5-106) and EH1.2 (purple, 346-448). One calcium atom (orange sphere) was included in each structure based on the coordination observed in canonical EF-hand motifs.

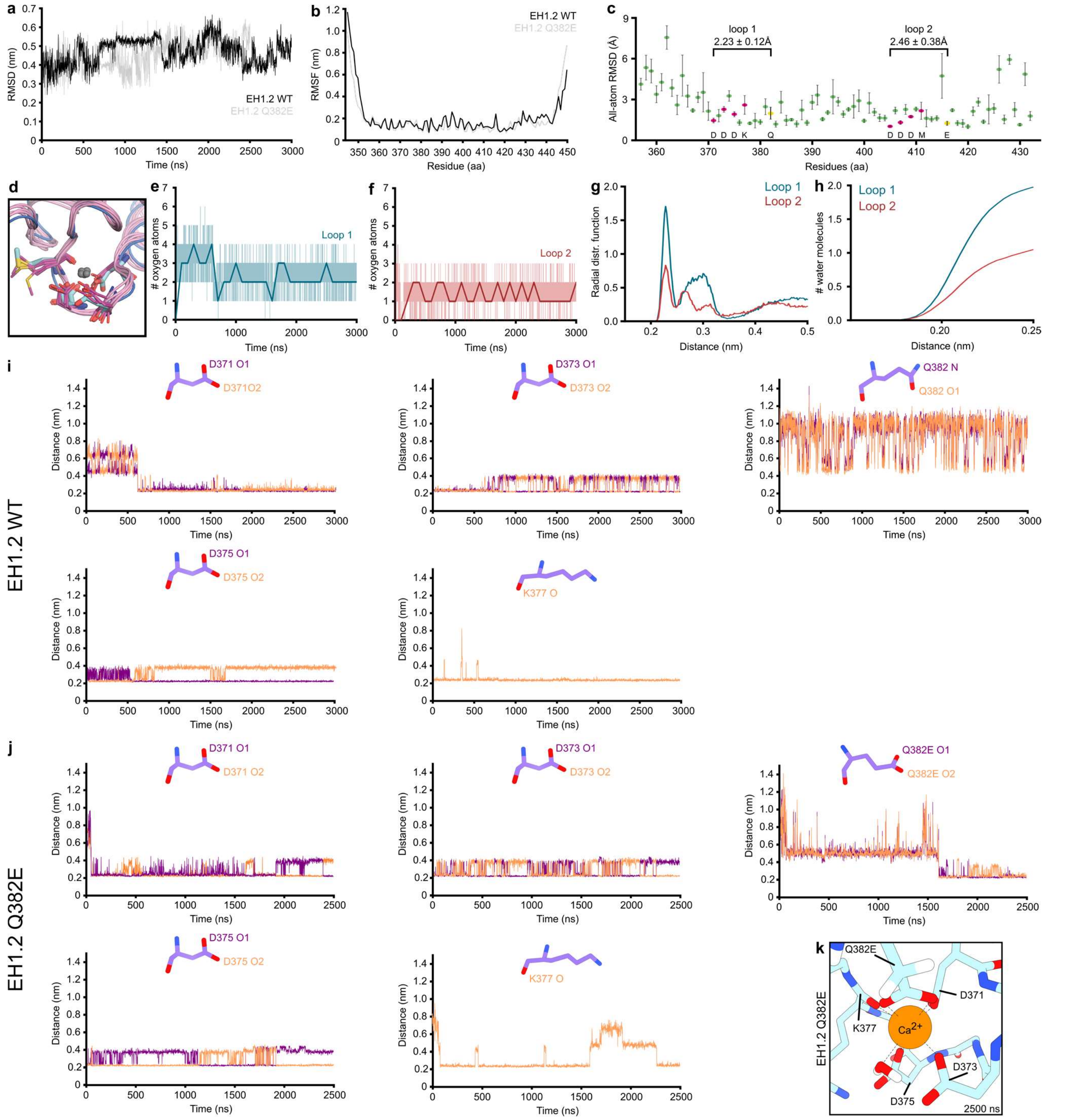

Supplementary Figure 2: A possible coordination of the first loop of EH1.2 can be obtained by all-atom molecular dynamics simulations.

a, Root mean square deviation (RMSD) of EH1.2 WT (black) and the Q382E mutant (grey) over the simulation time. **b**, Root mean square fluctuation (RMSF) of EH1.2 WT amino acid residues (black) and the Q382E mutant (grey) over the last 500ns of the simulation. The comparison of RMSF shows the flexibility of both protein termini for both WT and mutant structures. **c**, RMSD between NMR and five all-atom MD based structures. The majority of the Ca^{2+} coordinating residues are shown in pink. Residues Q382 and E416 are shown in yellow. RMSD values for loop 1 and loop 2 area shown above the indicated residues. Data are presented as mean values ± SD. **d,** Overlap of NMR (light blue) and five timepoints from the last 200 ns of the all-atom MD simulation (purple). Calcium is shown in grey. **e-f**, Number of oxygen atoms of water molecules within the 0.25 nm distance from the calcium atom in each EH1.2 loop over the all-atom MD simulation. The light color represents sampling every 100 ps, the dark color represents

sampling every 100 ns. **g**, Radial distribution function between Ca^{2+} and water molecules calculated over the last 500 ns of the all-atom MD simulation. **h**, The average number of water molecules within a particular distance calculated over the last 500 ns of the all-atom MD simulation. **i-j**, Oxygen to calcium (nitrogen and oxygen in the case of residue Q382) distance over the complete simulation of the residues potentially involved in Ca^{2+}-coordination. **k**, Cartoon representation of the first EF-hand loop of the all-atom MD structure of EH1.2 Q382E. Ca^{2+} is shown in orange. The extensive molecular dynamics simulation did not reveal any contribution of Q382 in the coordination of calcium. This is in contrast to the simulation with the mutated EH1.2 domain, where canonical calcium coordination and hence contribution of Q382E was observed (defined as a 2.5 Å distance between calcium and the depicted atom).

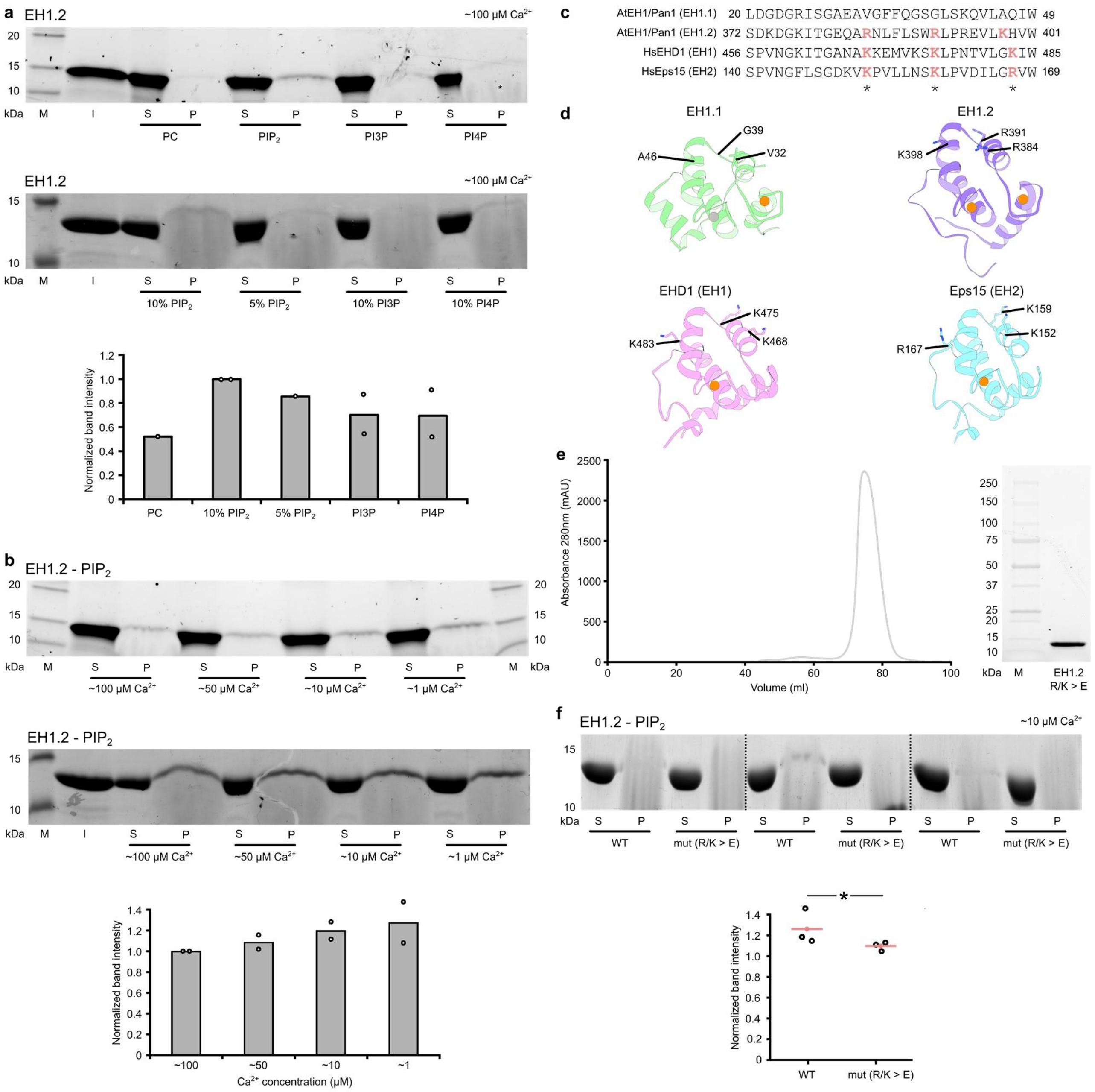

Supplementary Figure 3: The AtEH1/Pan1 EH domains are involved in lipid binding.

a, Liposome binding assay and corresponding quantification plot between EH1.2 and liposomes containing an equimolar mixture of PE and PC (indicated as PC in panel a) or a combination of PC/PE and different anionic phospholipids. I=Input, S=supernatant, P=pellet M=Marker. Both repetitions are shown. **b**, Liposome binding assay and corresponding quantification plot of EH1.2 with 10 % PIP_2 liposomes in the presence of different Ca^{2+} concentrations. I=Input S=supernatant, P=pellet M=Marker. Both repetitions are shown. **c**, Sequence alignment of the EH domains of AtEH1/Pan1 and EH domains shown by NMR to bind phosphoinositides. Conserved lysine and arginine residues are highlighted in pink. Residues shown to bind phosphoinositides by NMR[24] are indicated with an asterisk. **d**, Cartoon representation of EH1.1, EH1.2, EHD1 (2KSP), and Eps15 (1F8H) with residues highlighted in panel c, shown as sticks. **e**, SEC profile and gel of the purification of EH1.2 R/K > E. The elution profile was similar to the non-mutated form. The three mutated residues are highlighted in panel c. The experiment was performed twice with similar results. **f**, Liposome binding assay and corresponding quantification plot of EH1.2 and EH1.2 R/K > E, performed in triplicate. S=supernatant,

P=pellet. Statistical analysis was performed using a two-sided permutation t-test ($p=0.0118$). The bands in a and b are quantified and the values were normalized per gel (to the 10% PIP_2 band in panel a and to the 100 µM Ca^{2+} band in panel b) to allow comparison. Band intensities in panels a, b and f were quantified as average grey values measured in the pellet fraction, normalized to the average intensity of a similar sized region in the background. Quantifications of the panels a and b are made for the sake of keeping data representation comparable to the main display items.

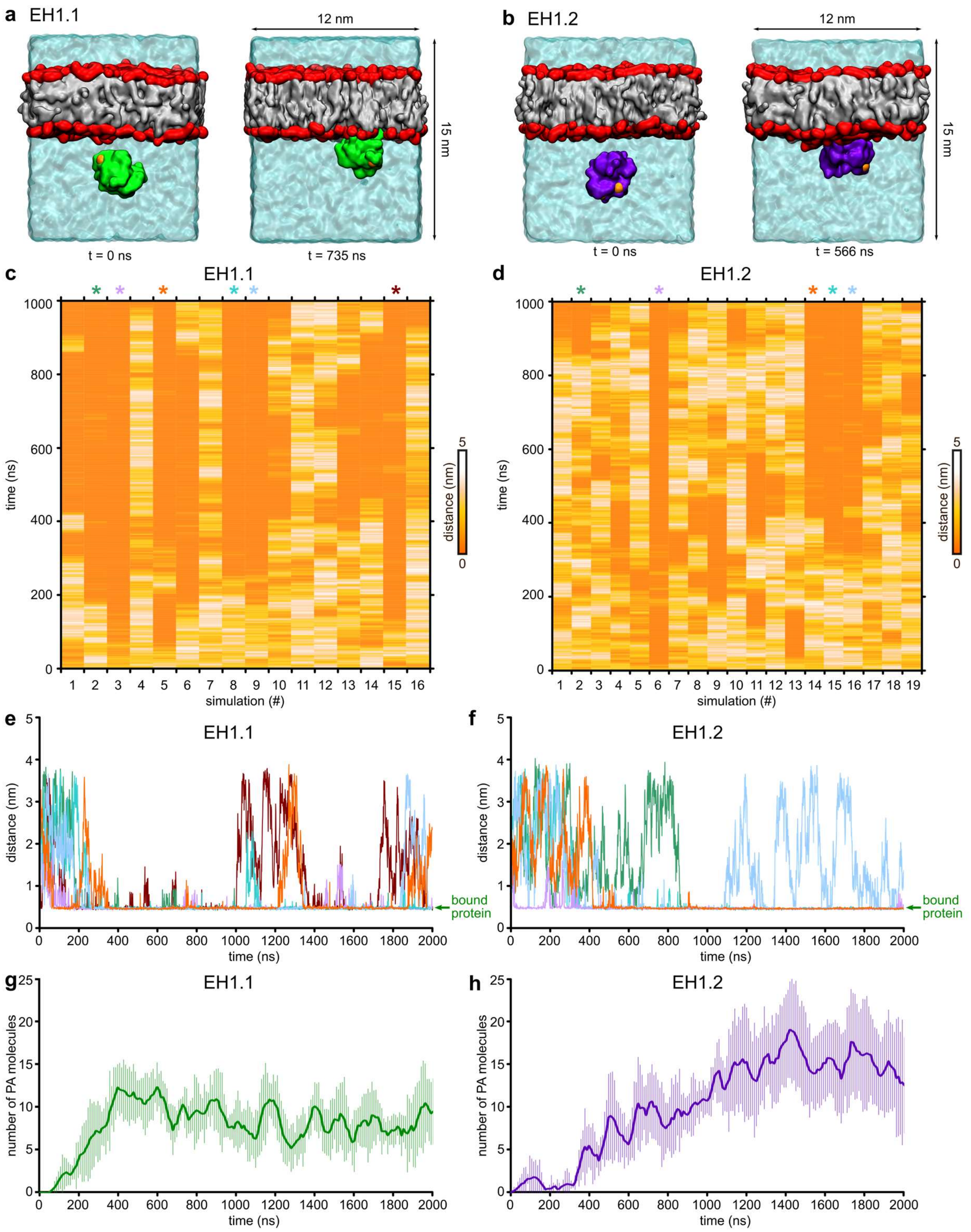

Supplementary Figure 4: MD simulation predicts that both AtEH1/Pan1 EH domains interact with phosphatidic acid (PA).

a-b, Representative snapshots of the CG-MD simulations of EH domains with a lipid bilayer containing 20% PA. Two timepoints are shown, the starting conditions (0 ns) and the membrane bound protein (time 735 ns for EH1.1 and time 566 ns for EH1.2). EH1.1 is colored in green, EH1.2 is in blue, acyl chains are grey, headgroup atoms are red, calcium atoms are orange and water molecules are transparent cyan. Sodium and chloride atoms are not shown for the sake of clarity. **c-d**, Progress of the coarse-grained MD simulations as the distance between the EH domain and the membrane over time shown for each replica. For both EH domains, several replicas with the long-

lived protein-membrane interactions could be observed, i.e. the protein was stably associated with the membrane for a major part of the simulation time (highlighted by asterisks). During 1 μs simulations, we observed that EH1.1 more readily formed contacts with the membrane than EH1.2, but we noticed recurring unbinding events in several replicates with the long-lived contacts between EH1.1 and the membrane. **e-f**, Replicates with the long-lived protein-membrane interactions were simulated up to 2 μs. Progress of the simulations is shown as the minimal distance between the protein and the lipid bilayer. The green arrow indicates the distance at which the protein would be bound to the membrane. Line colors correspond to the colors of asterisks in panel g and h. In the extended simulations, we again observed recurring unbinding/binding events for EH1.1 in all extended replicates. In contrast to EH1.1, in the majority of EH1.2 replicates, the protein associated closely with the membrane through the extended simulations.. **g-h**, Number of PA molecules within the 0.8 nm distance from the EH domain over simulation time. The dark trace represents a sliding average of the PA molecules smoothed over a 50 ns window. The light colored lines represent a standard error of mean for individual time points (10 ns). The EH1.2 domain, in average, coordinates a higher number of the PA molecules than the EH1.1 domain over the simulation time.

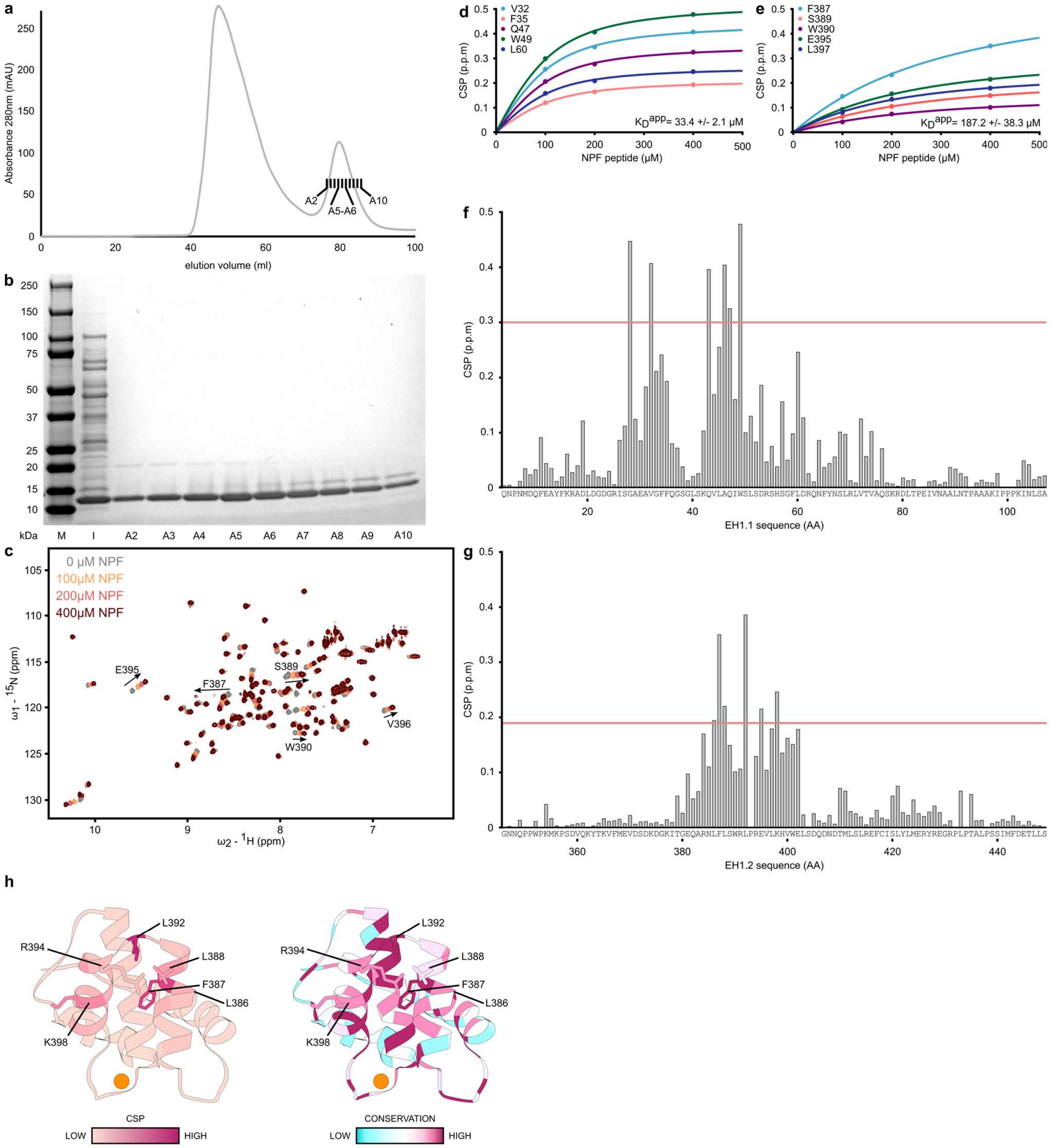

Supplementary Figure 5: Elucidation of the interaction between SCAMP5 and AtEH1/Pan1. a, Elution profile of EH1.1 W49A (Superdex 75 16/600 Hiload). **b**, Coomassie-blue SDS-PAGE (4-20%) analysis of the eluted fractions, shown in panel a, of the mutated EH1.1 domain (EH1.1 W49A). Coomassie SDS-PAGE of the fractions was performed once. **c**, ^{1}H-^{15}N-HSQC spectra of EH1.2 showing chemical shift perturbations with increasing amounts of SCAMP5 NPF peptide (grey to red). The highlighted residues were used for Kd measurements in panel e. **d-e**, NMR binding analysis of the interaction between EH1.1 (d) or EH1.2 (e) and the SCAMP5 N-terminal NPF peptide. The average dissociation constants (K_D) were estimated based on non-linear best fitting of the chemical shift perturbation (in ppm) of selected residues. **f-g**, Chemical shift perturbation per residue of EH1.1 and EH1.2 upon the addition of 400 μM double NPF peptide. The red line indicates the cut-off used in Fig. 3, panel d-e. **h**, Cartoon

representation of the structure of the NMR obtained EH1.2 structure (351-448) colored according to chemical shift perturbations (in ppm) (left) or according to Consurf colors (right). Six residues showing the large chemical shift perturbations are shown as sticks.

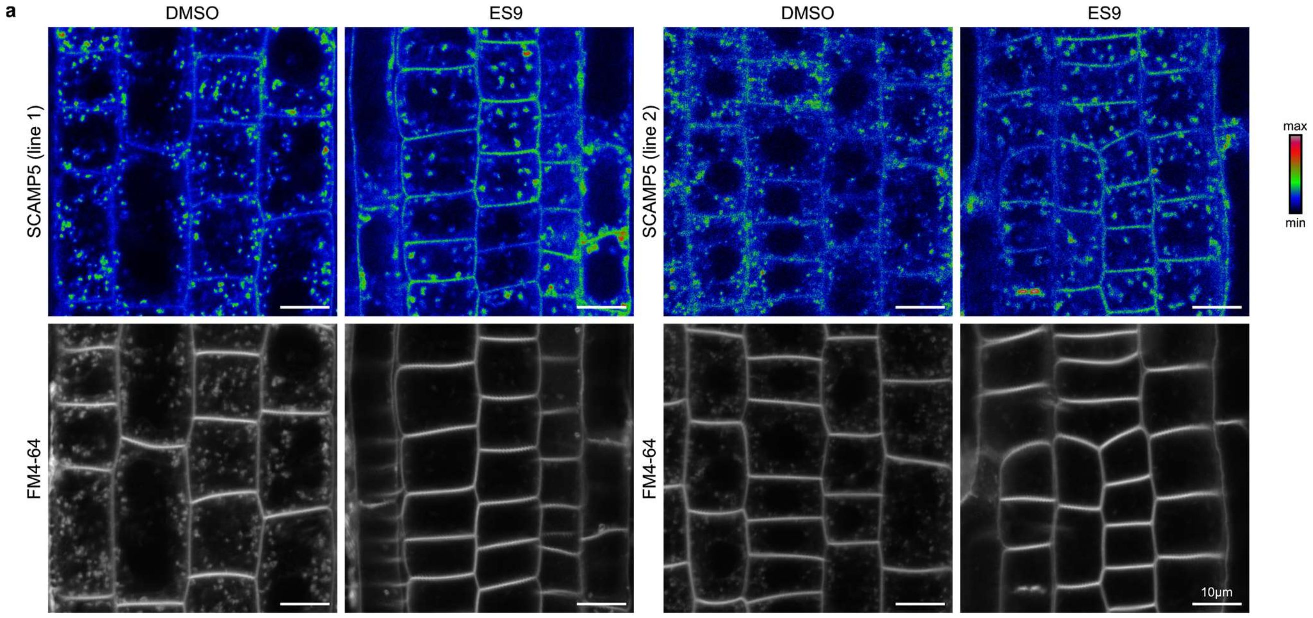

Supplementary Figure 6: Disruption of endocytosis by ES-9 results in accumulation of SCAMP5 at the PM.
a, Short term ES9 treatment of two independent SCAMP5-GFP lines results in SCAMP5 PM accumulation. DMSO was used as a control. The styryl dye FM4-64 was used as a marker to monitor the effect of ES9 on the endocytic uptake. Colors vary from red (max) to green to blue (min), to indicate the signal intensity. Scale bar indicates 10 µm.

Supplementary Table 1: X-ray refinement statistics for EH1.1

	EH1.1
**Data collection**	
Beam line	Petra III-P14
Wavelength (Å)	1.033
Space group	P 2_1 2_1 2_1
a,b,c (Å)	35.51 38.62 63.52
Resolution (Å)	33 - 1.55 (1.605 - 1.55)
Total reflections	177482 (7631)
Unique reflections	12827 (1058)
Multiplicity	13.8 (7.2)
Completeness (%)	97.15 (83.02)
Mean I/σ(I)	30.03 (4.69)
Wilson B-factor (Å^2)	15.68
R-merge	0.0515 (0.3554)
R-meas	0.05339 (0.3831)
R-pim	0.01378 (0.1387)
CC1/2 (%)	1 (0.962)
CC*	1 (0.99)
**Refinement**	
Reflections in refinement	12815 (1056)
Reflections used for R-free	642 (53)
Rwork	0.1449 (0.1944)
Rfree	0.1640 (0.2462)
CC(work)	0.968 (0.950)
CC(free)	0.962 (0.973)
Number of non-hydrogen atoms	889
Number of macromolecular atoms	792
Ligands	2
Solvent	95
Protein residues	198
RMS bonds (Å)	0.008
RMS angles (Å)	0.93
Ramachandran favored (%)	100
Ramachandran allowed (%)	0.00
Ramachandran outliers (%)	0.00
Rotamer outliers (%)	0.00
Clashscore	0.00
Average B-factor (Å^2)	22.05
macromolecules	20.70
ligands	19.56
solvent	33.32
Number of TLS groups	9

Statistics for the highest-resolution shell are shown in parentheses
Values reported by Phenix
Friedel pairs were treated as symmetry-related

Supplementary Table 2: NMR and refinement statistics for EH1.1 and EH1.2. Structural statistics of 20 NMR models

	EH1.1	EH1.2
NMR distance and dihedral restraints		
Distance restraints		
Total NOE	3127	2591
Intra-residue	570	588
Inter-residue	2557	2003
Sequential (|i – j| = 1)	732	615
Medium-range (1 < |i – j| < 5)	877	664
Long-range (|i – j| > = 5)	948	724
Total dihedral angle restraints	182	174
φ	91	87
ψ	91	87
Structure statistics		
Violations (mean ± s.d.)		
Distance restraints (Å)	0.021 ± 0.001	0.020 ± 0.001
Dihedral angle restraints (º)	0.243 ± 0.037	0.424 ± 0.046
Max. dihedral angle violation (º)	2.41	3.51
Max. distance constraint violation (Å)	0.363	0.322
Deviations from idealized geometry		
Bond lengths (Å)	0.013 ± 0.000	0.012 ± 0.000
Bond angles (º)	1.43 ± 0.030	1.408 ± 0.030
Impropers (º)	1.43 ± 0.044	1.338 ± 0.044
Average pairwise r.m.s. deviation* (Å)		
Heavy	0.63 ± 0.05	0.79 ± 0.07
Backbone	0.27 ± 0.04	0.41 ± 0.06
Ramachandran plot statistics* (%)		
Residues in most favoured regions	92.4	94.4
Residues in additionally allowed regions	7.6	5.6
Residues in generously allowed regions	0.0	0.0
Residues in disallowed regions	0.0	0.0

* Residues 6-105 of EH1.1 and residues 351-448 of EH1.2

Supplementary Table 3: Used primers

Construct	Forward primer	Reverse primer
6xHis-TEV-EH1.1	TAAGCACATATGGCACACCATCACCACCATCACGGGGAAAACCTGTATTTTCAGGGCGGGATGGCGGGTCAGAATCCTAACATGG	TGCTTACTCGAGTCAAGCTGAAAGATTAATTTTGGGAGGTGG
6xHis-TEV-EH1.2	TAAGCACATATGGCACACCATCACCACCATCACGGGGAAAACCTGTATTTTCAGGGCGGGGGAAATAATCAGCCTCCGTGGC	TGCTTACTCGAGTCAAGATAGCAGTGTTTCATCAAACATGATGC
6xHis-TEV-EH1.1 W49A	CAGATAGCGTCGCTTTCTGATCGGTCACACAG	GAAAGCGACGCTATCTGGGCGAGAACCTGC
pENTRY-FL_AtEH1-W49A	TCAGATAGCGTCGCTTTCTGATCGGTCACACAGTGG	CAGAAAGCGACGCTATCTGAGCGAGAACCTGCTTAGAC
pDONR221_P3-AtEH1-P2 (BIFC)	GGGGACAACTTTGTATAATAAAGTTGTAATGGCGGGTCAGAATCCTAACATGG	GGGGACCACTTTGTACAAGAAAGCTGGGTTGAAGGAGTTCCAGTTATCTGACCTTTTCG
pDONR221_P3-AtEH1stop-P2 (BIFC)	GGGGACAACTTTGTATAATAAAGTTGTAATGGCGGGTCAGAATCCTAACATGG	GGGGACCACTTTGTACAAGAAAGCTGGGTTTCAGAAGGAGTTCCAGTTATCTGACCTTTTCG

Supplementary Table 4: Ordered fragments

SCAMP5	TGTACAAAAAAGCAGGCTTAATGAATCGCCACCACGATCCCAATCCTTTCGATGAG GACGAAGAAATCGTCAATCCTTTTTCGAAAGGTGGTGGAAGGGTTCCTGCTGCATC TAGGCCAGTTGAATATGGTCAAAGCCTTGATGCTACTGTTGATATTCCATTGGATA ATATGAATGACTCTTCACAGAAACAGAGAAAGCTTGCTGACTGGGAAGCTGAGCTC AGGAAGAAAGAAATGGATATAAAGCGAAGAGAGGAAGCTATTGCTAAATTTGGTGT GCAGATAGATGATAAAAACTGGCCACCGTTTTTCCCAATCATACACCATGACATTG CTAAAGAGATACCAGTTCATGCACAAAAGCTGCAGTATCTGGCTTTCGCTAGTTGG TTAGGTATCGTTCTGTGTCTGGTATTCAATGTCATTGCAACGATGGTCTGCTGGAT TAAAGGCGGAGGTGTTAAAATCTTTTTCCTGGCCACAATATATGCATTGATCGGAT GTCCACTCTCTTATGTACTATGGTACAGGCCACTCTACCGAGCCATGAGGACTGAC AGTGCTTTGAAGTTTGGTTGGTTTTTCTTCACCTACTTGATTCACATTGGCTTCTG CATCGTTGCTGCCATCGCCCCTCCAATCTTTTTCCATGGAAAATCATTAACGGGTG TGCTTGCAGCAATTGATGTCATCTCAGACAGTTTATTAGCTGGGATCTTCTACTTT ATCGGATTCGGACTCTTCTGCTTGGAGTCACTGCTGAGTCTATGGGTTCTTCAGAA AATTTACCTCTACTTTAGGGGAAACAAGTACCCAGCTTTCTTGTACAA
ΔN SCAMP5	TGTACAAAAAAGCAGGCTTAATGGGTGGTGGAAGGGTTCCTGCTGCATCTAGGCCA GTTGAATATGGTCAAAGCCTTGATGCTACTGTTGATATTCCATTGGATAATATGAA TGACTCTTCACAGAAACAGAGAAAGCTTGCTGACTGGGAAGCTGAGCTCAGGAAGA AAGAAATGGATATAAAGCGAAGAGAGGAAGCTATTGCTAAATTTGGTGTGCAGATA GATGATAAAAACTGGCCACCGTTTTTCCCAATCATACACCATGACATTGCTAAAGA GATACCAGTTCATGCACAAAAGCTGCAGTATCTGGCTTTCGCTAGTTGGTTAGGTA TCGTTCTGTGTCTGGTATTCAATGTCATTGCAACGATGGTCTGCTGGATTAAAGGC GGAGGTGTTAAAATCTTTTTCCTGGCCACAATATATGCATTGATCGGATGTCCACT CTCTTATGTACTATGGTACAGGCCACTCTACCGAGCCATGAGGACTGACAGTGCTT TGAAGTTTGGTTGGTTTTTCTTCACCTACTTGATTCACATTGGCTTCTGCATCGTT GCTGCCATCGCCCCTCCAATCTTTTTCCATGGAAAATCATTAACGGGTGTGCTTGC AGCAATTGATGTCATCTCAGACAGTTTATTAGCTGGGATCTTCTACTTTATCGGAT TCGGACTCTTCTGCTTGGAGTCACTGCTGAGTCTATGGGTTCTTCAGAAAATTTAC CTCTACTTTAGGGGAAACAAGTACCCAGCTTTCTTGTACAA
EH1.2 R/K > E	TTTTGTTTAACTTTAAGAAGGAGATATACATATGGCACACCATCACCACCATCACG GGGAAAACCTGTATTTTCAGGGCGGGGGAAATAATCAGCCTCCGTGGCCAAAAATG AAACCATCCGATGTTCAGAAATACACAAAGGTATTTATGGAAGTTGATAGTGACAA GGATGGAAAAATCACTGGTGAGCAGGCGGAAAATCTATTTTTAAGCTGGGAGTTAC CCAGGGAGGTATTGGAGCATGTGTGGGAATTATCTGATCAGGATAATGATACTATG CTTTCTCTGAGGGAGTTCTGCATTTCATTGTATTTGATGGAGCGGTATAGAGAAGG CCGTCCTCTCCCGACCGCACTTCCTAGCAGCATCATGTTTGATGAAACACTGCTAT CTTGACTCGAGCACCACCACCACCACCACTGAGATCCGGCTGCTAACAAAGCCC
3x_EH1.1_GSlinker	TGTACAAAAAAGCAGGCTTAATGATGGCGGGTCAGAATCCTAACATGGATCAATTC GAGGCCTACTTCAAAAGAGCAGATTTAGACGGAGATGGTCGGATCAGTGGTGCCGA AGCTGTTGGATTTTTTCAAGGATCTGGTTTGTCTAAGCAGGTTCTCGCCCAGATAT GGTCGCTTTCTGATCGGTCACACAGTGGTTTCCTTGATCGGCAAAACTTTTATAAT TCTCTGAGACTTGTAACAGTTGCACAGAGCAAGAGAGATCTGACACCTGAGATTGT

	TAATGCAGCACTGAATACTCCTGCAGCAGCCAAAATACCACCTCCCAAAATTAATC TTTCAGCTGGTGGAGGGGGCTCAGGAGGAGGGGGTTCTGGTGGCGGCGGGTCTGCT GGTCAGAACCCGAATATGGATCAGTTTGAGGCCTACTTTAAGAGGGCGGACCTCGA CGGCGACGGACGGATTTCTGGTGCCGAGGCCGTCGGCTTCTTCCAAGGGTCCGGCC TTTCCAAGCAGGTCCTGGCGCAGATATGGTCCCTGTCCGACAGATCGCATTCAGGC TTCTTGGACAGACAGAACTTTTATAATTCACTTAGGCTCGTCACTGTCGCACAATC GAAGCGTGACCTCACCCCAGAAATCGTGAATGCAGCCCTCAATACGCCGGCTGCGG CGAAGATTCCGCCTCCGAAGATCAATCTCTCCGCCGGGGGCGGTGGCAGCGGAGGT GGAGGTAGTGGCGGAGGCGGATCAGCAGGACAAAACCCAAACATGGATCAGTTCGA GGCTTATTTTAAACGAGCAGATTTGGACGGTGATGGTAGAATATCCGGCGCTGAGG CAGTCGGGTTTTTCCAGGGATCCGGACTGTCAAAGCAAGTTTTGGCTCAAATATGG TCACTTTCCGACCGAAGTCATAGTGGGTTTCTTGACAGACAAAACTTTTACAATAG CTTAAGGCTTGTTACCGTTGCACAAAGCAAGAGGGATTTAACTCCAGAAATAGTTA ACGCCGCTTTAAATACACCTGCTGCCGCAAAGATTCCACCACCGAAGATTAACCTG TCCGCCTACCCAGCTTTCTTGTACAA
3x_EH1.2_G Slinker	TGTACAAAAAAGCAGGCTTAATGGAAATAATCAGCCTCCGTGGCCAAAAATGAAA CCATCCGATGTTCAGAAATACACAAAGGTATTTATGGAAGTTGATAGTGACAAGGA TGGAAAAATCACTGGTGAGCAGGCGAGGAATCTATTTTTAAGCTGGAGGTTACCCA GGGAGGTATTGAAGCATGTGTGGGAATTATCTGATCAGGATAATGATACTATGCTT TCTCTGAGGGAGTTCTGCATTTCATTGTATTTGATGGAGCGGTATAGAGAAGGCCG TCCTCTCCCGACCGCACTTCCTAGCAGCATCATGTTTGATGAAACACTGCTATCTG GTGGAGGGGGCTCAGGAGGAGGGGGTTCTGGTGGCGGCGGGTCTGGTAACAACCAA CCACCATGGCCGAAGATGAAACCAAGCGACGTTCAAAAGTACACAAAAGTTTTCAT GGAGGTCGATTCTGACAAGGATGGGAAGATTACTGGGGAACAAGCGAGGAACCTGT TTCTCAGCTGGCGTCTGCCAAGAGAGGTGCTGAAACATGTCTGGGAACTGAGCGAC CAGGATAATGATACCATGCTCTCTTTGAGGGAGTTCTGTATCTCTCTGTATCTCAT GGAGAGATACCGTGAAGGCAGGCCGCTTCCAACTGCTCTGCCCTCATCGATCATGT TTGACGAAACTCTCCTTAGCGGGGGCGGTGGCAGCGGAGGTGGAGGTAGTGGCGGA GGCGGATCAGGAAATAATCAACCCCCTTGGCCCAAGATGAAACCATCCGACGTCCA GAAGTATACCAAAGTGTTCATGGAAGTAGATAGTGATAAGGACGGTAAAATTACGG GTGAACAAGCACGGAACTTATTCTTAAGTTGGAGATTGCCACGTGAGGTGTTGAAG CACGTGTGGGAGCTTAGCGATCAAGATAACGATACCATGTTATCACTTAGAGAATT TTGTATATCTCTTTATCTTATGGAAAGATATAGAGAAGGTAGGCCTTTGCCCACAG CACTCCCTTCCTCAATTATGTTCGATGAAACTCTTCTCTCTTACCCAGCTTTCTTG TACAA

Supplementary Table 5: Mutants and transgenic lines used in this study.

Plant line	Background	Source
H3.3p::SCAMP5-GFP	H3.3p::AtEH1-mRuby in *eh1/pan1*(-/-)	This study
H3.3p::ΔN SCAMP5-GFP	Col-0	This study
H3.3p::SCAMP5-GFP	Col-0	This study
