# Supplementary material for: Distinct EH domains of the endocytic TPLATE complex confer lipid and protein binding
Source: Nat Commun. 2021 May 24;12:3050. doi: 10.1038/s41467-021-23314-6 (PMC8144573; doi:10.1038/s41467-021-23314-6)
Supplement: Supplementary file 3 — Description of Additional Supplementary Files [file 41467_2021_23314_MOESM3_ESM.pdf]

Description of Additional Supplementary Files

Supplementary Data 1: Instrument settings and data acquisition parameters for the Element XR SF-ICP-MS instrument.

Supplementary Data 2: MS Andromeda and Maxquant search parameters.

Supplementary Data 3: Mass spec data of peptidome profiling.

Supplementary Data 4: Full gels of lipid binding experiment.

Supplementary Data 5: Jalview compatible multiple alignment file and list of used species.

Supplementary Data 6: NMR/all-atom MD structure of EH1.2 and EH1.2 Q382E.
