# Supplementary material for: Distinct EH domains of the endocytic TPLATE complex confer lipid and protein binding
Source: Nat Commun. 2021 May 24;12:3050. doi: 10.1038/s41467-021-23314-6 (PMC8144573; doi:10.1038/s41467-021-23314-6)
Supplement: Supplementary file 10 — Reporting Summary [file 41467_2021_23314_MOESM10_ESM.pdf]

nature research

Corresponding author(s): Savvas N. Savvides, Kostantinos Tripsianes, Roman Pleskot, Daniel Van Damme

Last updated by author(s): 15/04/21

Reporting Summary

Nature Research wishes to improve the reproducibility of the work that we publish. This form provides structure for consistency and transparency in reporting. For further information on Nature Research policies, seeAuthors & Referees and theEditorial Policy Checklist .

Statistics

For all statistical analyses, confirm that the following items are present in the figure legend, table legend, main text, or Methods section.

n/a	Confirmed	
☐	☒	The exact sample size (*n*) for each experimental group/condition, given as a discrete number and unit of measurement
☐	☒	A statement on whether measurements were taken from distinct samples or whether the same sample was measured repeatedly
☐	☒	The statistical test(s) used AND whether they are one- or two-sided<br>*Only common tests should be described solely by name; describe more complex techniques in the Methods section.*
☒	☐	A description of all covariates tested
☐	☒	A description of any assumptions or corrections, such as tests of normality and adjustment for multiple comparisons
☐	☒	A full description of the statistical parameters including central tendency (e.g. means) or other basic estimates (e.g. regression coefficient) AND variation (e.g. standard deviation) or associated estimates of uncertainty (e.g. confidence intervals)
☐	☒	For null hypothesis testing, the test statistic (e.g. *F*, *t*, *r*) with confidence intervals, effect sizes, degrees of freedom and *P* value noted<br>*Give P values as exact values whenever suitable.*
☒	☐	For Bayesian analysis, information on the choice of priors and Markov chain Monte Carlo settings
☒	☐	For hierarchical and complex designs, identification of the appropriate level for tests and full reporting of outcomes
☒	☐	Estimates of effect sizes (e.g. Cohen's *d*, Pearson's *r*), indicating how they were calculated

Our web collection on statistics for biologists contains articles on many of the points above.

Software and code

Policy information about availability of computer code

Data collection	Bio-Rad Image Lab software package (version6.0.0 Build 25) was used for the collection of gel-images. Crystallographic and SAXS data were collected at Petra III (beamline P14). SEC-MALLS data were measured using a miniDAWN TREOS instrument (Wyatt) equipped with ASTRA 6.1. NMR data was collected at CEITEC Josef Dadok National NMR Centre on 850 MHz Bruker Avance III spectrometer equipped with 1H/13C/15N TCI cryogenic probehead with z-axis gradients. TXRF data was collected on a G.N.R. TX2000 total reflection X-ray fluorescence spectrometer. size exclusion chromatography was performed on a AKTA explorer with UNICORN 3.10 software. Mass spec data was recorded using a Q Exactive mass spectrometer (Thermo Fisher). BLI data was recorded using an Octet RED96 instrument in combination with Octet Data acquisition software V12 (FortéBio). Confocal images were recorded on a Leica SP8X system (LASX, different versions 2019-2020) and on a PE UltraView spinning disk microscopy using the Volocity software package (version 6.5.1). Calcium determination was accomplished using a Thermo Scientific Element XR (Bremen, Germany) single-collector sector-field inductively coupled plasma-mass spectrometer (SF-ICP-MS).
Data analysis	Software used to process, analyze and validate the crystallographic data was provided by the XDS (BUILD20180126), the CCP4 package version 7.0 (including AIMLESS, PHASER and PARROT), PHENIX version 1.12 (including phenix.refine and phenix.xtriage), autoBuster version 2.10.3 (Global Phasing Ltd.) and the PDB_REDO server. Structures were (re)build using Coot (version 0.8.8); NMR structure calculations were performed with the help of the 4D chains algorithm in combination with Cyana 3.0 and validated using PSVS v1.4. ImageJ version 1.52p (Fiji, https://fiji.sc/); XRF data were fitted using the AXIL software package; Molecular dynamics simulations were performed in GROMACS 5.1.2 package;R studio was used for statistical analysis (version 1.4.1103) (http://www.R-project.org); MaxQuant (version 1.6.4.0) and Perseus (version 1.6.1.1) were used for MS analysis; SEC-MALS results were analyzed using Astra 6.1 (Wyatt Technology). BLI kinetic data was analyzed using GraphPad Prism 5. Structural superpositions were made with Chimera (version 1.13.1). Secondary structure of protein models was assigned using the DSSP web server (version 3.0); Images were made using Inkscape (version1.0.1).

For manuscripts utilizing custom algorithms or software that are central to the research but not yet described in published literature, software must be made available to editors/reviewers. We strongly encourage code deposition in a community repository (e.g. GitHub). See the Nature Research guidelines for submitting code & software for further information.

Data

Policy information about availability of data

All manuscripts must include a data availability statement. This statement should provide the following information, where applicable:

- Accession codes, unique identifiers, or web links for publicly available datasets
- A list of figures that have associated raw data
- A description of any restrictions on data availability

The authors declare that all data supporting the findings of this study are available within the article and its Supplementary information files or from the corresponding authors upon reasonable request.
The structural data that support the findings of this study are available in the Protein Data Bank with the accession codes 6YIG (doi: 10.2210/pdb6YIG/pdb), 6YEU (doi:10.2210/pdb6YEU/pdb) and 6YET(doi: 10.2210/pdb6YET/pdb). Already published protein structures were used in this study to allow comparison. The following structures were used 2QPT (doi: 10.2210/pdb2QPT/pdb), 1CLL (doi:http://doi.org/10.2210/pdb1CLL/pdb), 2KSP (doi: 10.2210/pdb2KSP/pdb), 1F8H (doi: 10.2210/pdb1F8H/pdb).

Field-specific reporting

Please select the one below that is the best fit for your research. If you are not sure, read the appropriate sections before making your selection.

☒ Life sciences ☐ Behavioural & social sciences ☐ Ecological, evolutionary & environmental sciences

For a reference copy of the document with all sections, see nature.com/documents/nr-reporting-summary-flat.pdf

Life sciences study design

All studies must disclose on these points even when the disclosure is negative.

Sample size	No statistical method was used to determine sample size. Sample sizes were chosen based on our experience. At least two independent transgenic lines were used for every relevant study and at least two or three repetitions were performed for every study that was quantified. The amount of repetitions is clearly indicated in the figure legeds.
Data exclusions	No data were excluded from this study.
Replication	All experiments were performed independently at least twice and were quantification was applied all studies were performed in duplicate or triplicate.
Randomization	Randomization is not applicable to the study, as it does not involve clinical participants, assignment to experimental groups, or subjective analysis.
Blinding	Blinding was not implemented in this study as the experiments were performed without prior knowledge of the outcome. Key results were independently observed by two researchers.

Reporting for specific materials, systems and methods

We require information from authors about some types of materials, experimental systems and methods used in many studies. Here, indicate whether each material, system or method listed is relevant to your study. If you are not sure if a list item applies to your research, read the appropriate section before selecting a response.

Materials & experimental systems

n/a	Involved in the study
☒	☐ Antibodies
☒	☐ Eukaryotic cell lines
☒	☐ Palaeontology
☒	☐ Animals and other organisms
☒	☐ Human research participants
☒	☐ Clinical data

Methods

n/a	Involved in the study
☒	☐ ChIP-seq
☒	☐ Flow cytometry
☒	☐ MRI-based neuroimaging
